# Ethylene-Related Gene Expression Networks in Wood Formation

**DOI:** 10.3389/fpls.2018.00272

**Published:** 2018-03-14

**Authors:** Carolin Seyfferth, Bernard Wessels, Soile Jokipii-Lukkari, Björn Sundberg, Nicolas Delhomme, Judith Felten, Hannele Tuominen

**Affiliations:** ^1^Department of Forest Genetics and Plant Physiology, Umeå Plant Science Centre, Swedish University of Agricultural Sciences, Umeå, Sweden; ^2^Department of Plant Physiology, Umeå Plant Science Centre, Umeå University, Umeå, Sweden

**Keywords:** ethylene signaling, secondary growth, wood development, co-expression network, EIN3, ERF

## Abstract

Thickening of tree stems is the result of secondary growth, accomplished by the meristematic activity of the vascular cambium. Secondary growth of the stem entails developmental cascades resulting in the formation of secondary phloem outwards and secondary xylem (i.e., wood) inwards of the stem. Signaling and transcriptional reprogramming by the phytohormone ethylene modifies cambial growth and cell differentiation, but the molecular link between ethylene and secondary growth remains unknown. We addressed this shortcoming by analyzing expression profiles and co-expression networks of ethylene pathway genes using the AspWood transcriptome database which covers all stages of secondary growth in aspen (*Populus tremula*) stems. *ACC synthase* expression suggests that the ethylene precursor 1-aminocyclopropane-1-carboxylic acid (ACC) is synthesized during xylem expansion and xylem cell maturation. Ethylene-mediated transcriptional reprogramming occurs during all stages of secondary growth, as deduced from AspWood expression profiles of ethylene-responsive genes. A network centrality analysis of the AspWood dataset identified *EIN3D* and 11 *ERFs* as hubs. No overlap was found between the co-expressed genes of the *EIN3* and *ERF* hubs, suggesting target diversification and hence independent roles for these transcription factor families during normal wood formation. The *EIN3D* hub was part of a large co-expression gene module, which contained 16 transcription factors, among them several new candidates that have not been earlier connected to wood formation and a VND-INTERACTING 2 (VNI2) homolog. We experimentally demonstrated *Populus EIN3D* function in ethylene signaling in *Arabidopsis thaliana*. The *ERF* hubs *ERF118* and *ERF119* were connected on the basis of their expression pattern and gene co-expression module composition to xylem cell expansion and secondary cell wall formation, respectively. We hereby establish data resources for ethylene-responsive genes and potential targets for EIN3D and ERF transcription factors in *Populus* stem tissues, which can help to understand the range of ethylene targeted biological processes during secondary growth.

## Introduction

Woody tissue serves as plant stabilizing material, in nutrient storage and distribution of water and minerals. It is produced by the activity of the vascular cambium which undergoes periclinal cell divisions to produce secondary xylem (i.e., “wood”) inwards and secondary phloem outwards in the stem (Mellerowicz et al., 2001; Zhang et al., 2014). Xylem cells undergo four main phases of differentiation to achieve functional specialization; cell division, cell expansion (elongation and radial enlargement), secondary cell wall thickening (involving deposition of cellulose, hemicellulose, cell wall proteins, and lignin) and programmed cell death. Recent advances in genome sequencing of both gymnosperm and angiosperm tree species provide novel tools to dissect the complex nature of these phases (Tuskan et al., 2006; Birol et al., 2013; Nystedt et al., 2013; Myburg et al., 2014; Salojärvi et al., 2017). Especially powerful are the transcriptomic and proteomic datasets that have been generated in aspen (*Populus tremula*) and Norway spruce (*Picea abies*) (Obudulu et al., 2016; Bygdell et al., 2017; Jokipii-Lukkari et al., 2017; Sundell et al., 2017). Their high spatial resolution within the woody tissues allows analyses in specific phases of wood formation, from cell division to cell death. Transcriptomic datasets from aspen and Norway spruce (*Picea abies*) are also easily accessible in the form of the AspWood and NorWood databases (Jokipii-Lukkari et al., 2017; Sundell et al., 2017).

The activity of the vascular cambium as well as secondary xylem differentiation is regulated by various plant hormones. Whilst cytokinins control cambial cell division activity, auxin is related to both cambial cell proliferation and xylem differentiation (for a recent review see Zhang et al., 2014). Also gibberellins and brassinosteroids control specific aspects of xylem differentiation (Eriksson et al., 2000; Caño-Delgado et al., 2004). The gaseous plant hormone ethylene stimulates cambial growth and induces typical features of reaction wood (such as G-layer formation) when applied exogenously (Brown and Leopold, 1973; Du and Yamamoto, 2007; Love et al., 2009; Felten et al., 2018). Genetic evidence supports the role of ethylene as a stimulator of cambial activity in *Arabidopsis thaliana* (Etchells et al., 2012) and in gravitropically stimulated *Populus* trees (Love et al., 2009). A number of ethylene biosynthetic enzymes and transcription factors (TFs) have been shown to be expressed in woody tissues of *Populus* trees (Andersson-Gunnerås et al., 2003; Vahala et al., 2013), but description of the whole ethylene pathway in this context is lacking.

**Figure 1:**
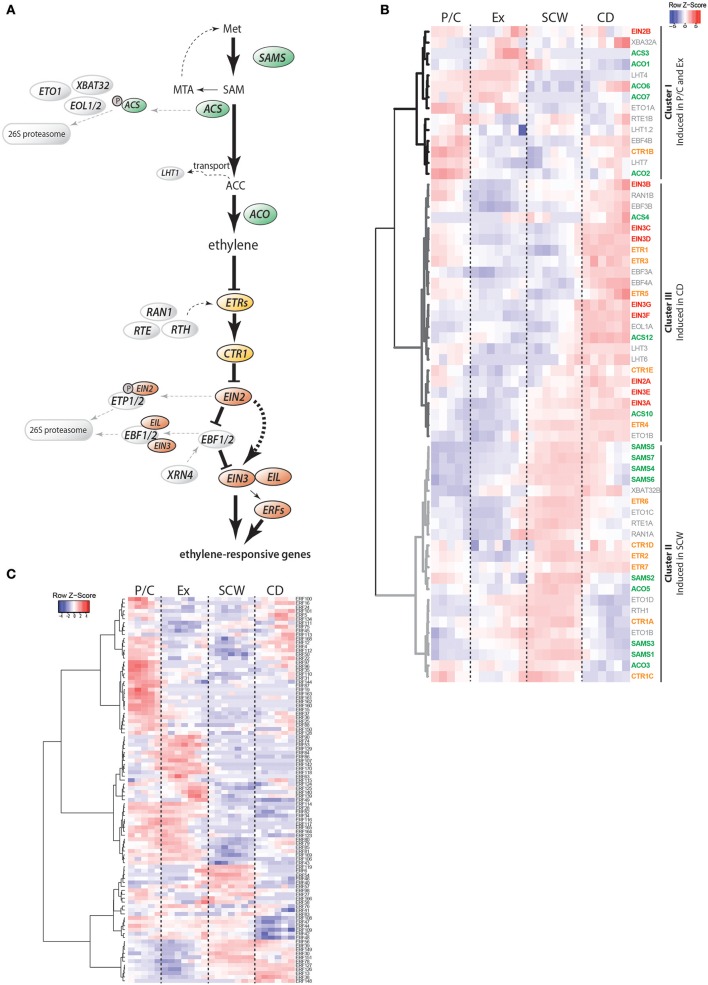
Stem expression pattern of ethylene pathway genes. **(A)** Schematic representation of the current model of ethylene biosynthesis and signaling. Ethylene is the end product of subsequent conversion from methionine by S-AdoMet synthetases (SAMS), ACC synthases (ACSs) and ACC oxidases (ACOs). Proteasome-mediated degradation of ACSs and transport of ACC are two mechanisms for plants to adjust cellular ethylene levels. Perception of ethylene is achieved through ER membrane-localized receptors (ETRs) that repress ethylene signaling in the absence of ethylene. Receptor activity is modulated by RAN1, RTE1 and RTH. In the absence of ethylene, ETRs prevent signaling and transcriptional reprogramming by activating the receptor-associated kinase CTR1. CTR1 controls activation of EIN2 in a phosphorylation-dependent manner. Once ethylene is bound to the receptors, the inhibitory activity of CTR1 is blocked, leading to cleavage of the EIN2 C-terminus which is now able to prevent proteasome-mediated degradation of the transcription factors EIN3 and EIL1. Downstream of EIN3, other transcription factors such as ethylene response factors (ERFs), contribute to transcriptional reprogramming of ethylene-responsive genes. Proteins are divided into biosynthesis (green), receptors and signaling (orange), transcriptional reprogramming (red) and regulatory (gray) components. Met, methionine; SAM, S-adenosylmethionine; MTA, Methylthioadenosine; ACC, 1-Aminocyclopropane-1-carboxylic acid; SAMS, S-adenosylmethionine synthetase; ACS, ACC synthase; MPK, Mitogen-activated protein kinase; ETO, Ethylene overproducer; EOL, Ethylene overproducer like; XBAT, XB3 ortholog; LHT, Lysine histidine transporter; ACO, ACC oxidase; ETR, Ethylene receptor; CTR, Constitutive triple response; RTE, Reversion-to-ethylene sensitivity; RTH, RTE-homolog; RAN, Resistance to antagonist; EIN, Ethylene insensitive; EIL, Ethylene-insensitive like; ETP, EIN2 targeting protein; EBF, EIN3-binding F-Box protein; XRN, Exoribonuclease; ERF, Ethylene response factor; P/C, Phloem/Cambium; Ex, Expanding xylem; SCW, Secondary cell wall formation; CD, Cell death **(B)** Heatmap showing the expression profiles of ethylene pathway genes in AspWood. Expression values are scaled per gene; red indicates gene expression higher than the associated cluster's average expression, while blue indicates gene expression lower than the average. Non-scaled expression values are shown in Supplementary Figure S3 and listed in Supplementary Table S2. **(C)** Expression profiles of *ERFs* in AspWood. Scaled expression values are shown (non-scaled expression values are listed in Supplementary Table S2).

Ethylene is the product of stepwise conversion of the amino acid methionine to S-adenosylmethionine (S-AdoMet) by S-AdoMet synthetase (SAM), then to 1-aminocyclopropane-1-carboxylate (ACC) by ACC synthase (ACS) and finally to ethylene by ACC oxidase (ACO) (Figure 1A; recently reviewed in Vanderstraeten and Van Der Straeten, 2017). Perception of ethylene occurs at the endoplasmic reticulum (ER) by a family of membrane-bound ethylene receptors (ETR). The ethylene receptors associate with the serine/threonine protein kinase CONSTITUTIVE TRIPLE RESPONSE 1 (CTR1) (reviewed in Merchante et al., 2013; Xu and Zhang, 2014). In the absence of ethylene, the ETR-CTR1 complex represses downstream ethylene signaling, while in the presence of ethylene ETR dissociates from CTR1 leading to activation of ethylene signaling by the downstream component ETHYLENE INSENSITIVE 2 (EIN2). Hereby, EIN2 is cleaved (Wen et al., 2012) and the C-terminus of EIN2 (EIN2-C) prevents proteasome-mediated degradation of the TFs ETHYLENE INSENSITIVE 3 (EIN3) and EIL1 (EIN3-like 1) by suppressing function of the two F-box proteins EIN3-BINDING F-BOX (EBF)1 and EBF2 (Li et al., 2015; Merchante et al., 2015). An additional signal transduction mechanism is created by relocalization of EIN2-C into the nucleus and interaction with ENAP1 (EIN2 nuclear associated protein 1), which triggers EIN3-mediated transcriptional reprogramming due to EIN2-C regulated histone acetylation (Zhang et al., 2016, 2017). Although multiple EIN3-like proteins exist in *A. thaliana*, knock-out of *EIN3* alone is sufficient to perturb ethylene signaling (Chao et al., 1997). A second layer of transcriptional regulation of ethylene-responsive genes consists of TFs belonging to the large family of ETHYLENE RESPONSE FACTORs (ERFs) (Licausi et al., 2013; Müller and Munné-Bosch, 2015). Most of our current knowledge about the ethylene pathway comes from *A. thaliana*. In woody species like *Populus*, only ERFs are well described (Vahala et al., 2013; Wang et al., 2014; Yao et al., 2017). Better understanding of the composition, function and molecular regulation of the ethylene pathway components and downstream gene targets in woody species is needed to reveal the role of ethylene in wood formation.

Here, we aimed to elucidate the ethylene pathway in *Populus* trees by identifying the ethylene signaling components and putative downstream targets, and assaying their expression during various stages of wood formation. For this purpose, we utilized two datasets: first, the recently published AspWood database, encompassing a near-tissue level transcriptome data from the vascular cambium and its derivative tissues (Sundell et al., 2017). Exploring this resource enabled us to reveal how ethylene biosynthesis and signaling components are expressed during cambial growth and normal wood formation. In addition, we used RNA-Seq libraries obtained from wild type and ethylene-insensitive transgenic *Populus tremula* × *tremuloides* trees treated with the ethylene precursor ACC (Felten et al., 2018), thus representing rapid transcriptomic changes upon elevated ethylene signaling. This latter dataset allowed us to identify potential ethylene regulated target genes and to pinpoint the phases where ethylene-mediated transcriptional reprogramming occurs during normal wood formation. Since *Populus tremula* and *Populus tremula* × *tremuloides* were used for the AspWood and ACC-dataset, respectively, we decided to map the libraries from the latter one to the *Populus tremula* genome. Our analyses showed that the expression of *ACC synthases* is under strict spatial control in woody tissues, suggesting that ACC is synthesized specifically during xylem cell expansion and late maturation. Ethylene signaling, however, seems to occur in a more ubiquitous manner. A centrality analysis of the gene network led to the identification of key genes (so-called “hubs”) in the wood transcriptome, among them *EIN3D* and 11 *ERF*s. The majority of the hubs have not been previously connected to wood formation. Altogether, our study provides a comprehensive data resource on the expression of ethylene pathway genes and potential ethylene-regulated targets in *Populus*, and clues on their functional significance in wood formation.

## Results and discussion

### *Populus* genome displays unique features within the ethylene pathway gene families

We performed phylogenetic analyses to identify homologs within the ethylene pathway gene families in the *Populus trichocarpa* (*Pt*), *Picea abies* (*Pa*) and *Arabidopsis thaliana (At)* genomes (Supplementary Figure S1, Supplementary Table S1). *P. trichocarpa* was used as it represents the *de facto* reference genome within the highly conserved *Populus* genus, while *Picea abies* was included to strengthen the identification of wood-related gene family members. For almost all gene families in the ethylene pathway, except the ethylene receptor family, *P. abies* genes clustered apart from *P. trichocarpa* and *A. thaliana*, as expected due to its evolutionary ancestry. Our analyses further revealed that all gene families in the ethylene pathway are expanded in the *P. trichocarpa* genome, in accordance with the rather recent whole genome duplication (Tuskan et al., 2006).

We observed expansion of the *CTR1* family in the *P. trichocarpa* genome, with five copies present compared to a single copy in the *A. thaliana* and spruce genomes (Supplementary Figures S1E, S2). Apart from three full-length *CTR1* isoforms, we found additional *Populus* genes encoding for either the N- or the C-terminus alone, *CTR1D* (*Potri.016095700*) and *CTR1E* (*Potri.016095800*), respectively (Supplementary Figure S2). In the absence of ethylene, the CTR1 N-terminus binds to the receptors, while the C-terminus containing the kinase domain, prevents transcription of ethylene-regulated genes by triggering degradation of EIN2 (Clark et al., 1998; Huang et al., 2003; Ju et al., 2012). Mutations in either the N- or C-terminus of CTR1 inhibits its suppressor function, indicating that both are essential to prevent constitutive ethylene signaling (Huang et al., 2003). A close relative of *P. trichocarpa, Salix purpurea*, encodes two genes that span the full-length *CTR1* sequence (*SapurV1A.0208s0240*; *SapurV1A.0208s0220*) and a third one (*SapurV1A.3019s0020*) which, similar to the *Populus CTR1E*, mainly covers the C-terminus (Supplementary Figure S2). The functional relevance of this CTR1 bifurcation and its presence in other species needs further investigation.

### Spatial expression pattern of *ACC synthases* suggests that ACC synthesis occurs during xylem cell expansion and maturation

To identify sites of ethylene biosynthesis and ethylene-mediated transcriptional reprogramming, we surveyed transcript abundances for ethylene pathway genes using AspWood (Supplementary Figure S3, Figure 1B). AspWood represents a high-spatial-resolution transcriptomic database generated from RNA-Seq analysis of a longitudinal cryosection series from the stem, encompassing phloem/cambium differentiation (P/C), xylem cell expansion (Ex), secondary cell wall (SCW) formation and programmed cell death (CD) (Sundell et al., 2017). Hierarchical clustering of the ethylene pathway genes in AspWood (Figure 1B) identified three main clusters: Cluster I includes genes that are highest expressed in the phloem, cambium and expanding xylem; Cluster II includes genes that show highest expression during SCW formation; and Cluster III, comprised of genes that are induced during xylem maturation when xylem cells undergo CD.

Four of the ten *Populus ACS* genes (Supplementary Figure S1), *ACS3, ACS4, ACS10*, and *ACS12*, show expression profiles in AspWood (Figure 1B). Based on protein sequence similarity, the *Populus* ACS3 and ACS4 are most similar to the enzymatically active *A. thaliana* ACSs (Yamagami et al., 2003), while the *Populus* ACS10 and ACS12 are homologous to the enzymatically inactive ACSs in *A. thaliana* (ACS10 and ACS12) (Supplementary Figure S1B). The *Populus ACS3* and *ACS4* had their highest expression in expanding xylem and during late xylem maturation (late CD zone), respectively (Supplementary Figure S3B, Supplementary Table S2). Genes encoding the potentially enzymatically inactive *Populus* ACSs, *ACS10*, and *ACS12*, had an overall high expression throughout the woody tissues (Supplementary Figure S3B). Similarly, expression analysis of *Pinus taeda* (*Pit*) xylem scrapings revealed low expression of *PitACS1* while the *PitACS1s* splice variant, lacking the tyrosine residue conserved in the active ACSs, was expressed more than three-fold in comparison (Barnes et al., 2008). The tomato homolog of the enzymatically inactive *A. thaliana ACS12* also showed highest expression within the *ACS* family in xylem tissues (Vanderstraeten and Van Der Straeten, 2017). Taken together, the expression of genes encoding the potentially enzymatically active *Populus* ACSs in the zones of xylem expansion and late maturation support these as the sites of ACC biosynthesis in the woody tissues. The constitutive expression of the genes encoding the potentially inactive ACSs support function of also these enzymes during wood formation.

ACC is distributed within the plant by transporters such as the recently identified LYSINE HISTIDINE TRANSPORTER 1 (LHT1) (Shin et al., 2015). Seven *LHT* genes were identified in *Populus*, with peaks in their expression in the P/C, Ex, and CD zones (Figure 1B). Thus, it may be that ACC is synthesized by the activity of the *Populus* ACS3 and ACS4 in the expanding xylem and late CD, from where it is transported to the other zones by the LHT transporters. This would be in line with the previously reported ubiquitous lateral distribution pattern of ACC throughout the woody tissues of *Populus* stems (Andersson-Gunnerås et al., 2003). We also observed that even though the six different *Populus ACOs* have very different expression patterns, they together provide *ACO* transcripts across all zones of wood formation (Supplementary Figures S1, S3C). From this follows that ethylene is potentially synthesized at any stage of xylem differentiation. Taken together, our results support that ACC biosynthesis occurs in the stages of xylem expansion and xylem maturation. Future work is needed to elucidate whether there are mechanisms, such as transport of ACC, which would allow lateral transport of ACC and hence production of ethylene in a ubiquitous manner during wood formation.

### Ethylene signaling components and TFs are expressed throughout all phases of wood formation

We analyzed whether ethylene receptors and the downstream signaling components (see Supplementary Figure S1) show specific expression patterns during wood formation by investigating their expression in the AspWood database. The different isoforms of both *ETR* and *CTR1* families were highly expressed during either SCW formation (*ETR6, CTR1A, CTR1C*) or CD (*ETR1, ETR3, ETR5*), or during both (*ETR2, ETR4, ETR7, CTR1D, CTR1E*) (Figure 1B; Cluster II and III). *CTR1B* forms an exception as it is highest expressed in P/C and late CD. As the simultaneous presence of ethylene receptors and CTR1 acts to suppress ethylene signaling it is possible, on the basis of their predominant expression during SCW formation, that downstream ethylene signaling is suppressed in this phase of xylem differentiation during normal wood formation.

Ethylene perception at the ER membrane is linked to gene regulation by the joint activity of the EIN2, EBF and EIN3 family members. The *Populus EIN2* genes were constitutively expressed during secondary growth in the AspWood database (Supplementary Figure S3F, Supplementary Table S2). In contrast, the EBF F-box protein encoding genes showed high expression in P/C, a slight increase during SCW formation and the highest expression during xylem maturation (CD zone) (Figure 1B, Supplementary Figure S3H). The *EIN3* family in *Populus* consists of seven genes (Supplementary Figure S1G) which all had, similar to the *EBFs*, their highest expression in P/C and CD (Figure 1B, Supplementary Figure S3G). Co-expression of the *EIN3* and *EBF* genes is consistent with the reported EIN3-mediated induction of *EBF2* expression in *A. thaliana* (Konishi and Yanagisawa, 2008). The high expression of the *Populus EIN3s* in P/C and during xylem maturation supports enhanced ethylene signaling during these phases of normal wood formation. However, even though *EIN3* expression levels have been causally related to ethylene function in *A. thaliana* (Zhong et al., 2009), EIN3 is also regulated through posttranscriptional regulation (Binder et al., 2007). Functional studies are therefore needed to unequivocally demonstrate the function of the *Populus* EIN3 proteins in wood formation.

*Populus* ERFs are divided into 10 subgroups according to protein sequence similarity to *A. thaliana* ERFs (Vahala et al., 2013). Of the 170 *ERFs* of *P. trichocarpa*, 98 *ERF*s are expressed in AspWood (Figure 1B). The *Populus ERFs* cluster in groups with zone-specific peaks in their expression profile [e.g., *ERF87* (P/C), *ERF118* (Ex), *ERF119* (SCW), *ERF57* (Ex + SCW), or *ERF126* (SCW + CD)] (Figure 1C). Misregulated expression patterns and/or altered expression levels of *ERF*s have been shown to affect tree growth (Vahala et al., 2013). An important task for the future is, however, to identify which part of the *ERF* gene family is related to ethylene signaling and what biological processes are targeted by the various ERFs.

### Ethylene-mediated transcriptional reprogramming occurs in all developmental zones

**Figure 2:**
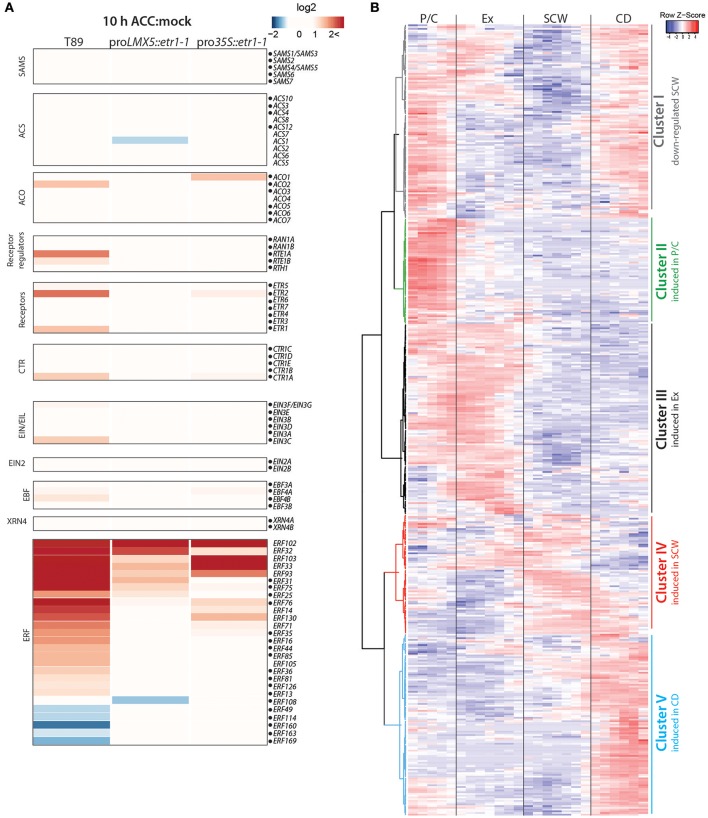
Transcriptional regulation of ethylene-responsive genes occurs in all developmental zones. **(A)** Expression of ethylene pathway genes in *Populus* trees treated with either water (mock) or 100 μM ACC for 10 h. RNA-Seq data was retrieved from Felten et al. (2018). Heatmap shows log2-values of gene induction/suppression between mock and ACC treatment in wild type (T89) and two ethylene-insensitive trees (pro*LMX5::etr1-1* and pro*35S::etr1-1*). RNA-Seq reads were mapped to the *P. tremula* genome (corresponding *P. trichocarpa* gene identities are listed in Supplementary Table S3). Dots mark genes that also have expression profiles in AspWood. In case of the large *ERF* family we chose to only present *ERFs* with at least a two-fold expression change between mock and ACC treatment in any of the three genotypes. **(B)** Hierarchical clustering and scaled expression profiles of ethylene-responsive genes from AspWood (*n* = 458; Supplementary Table S3). Ethylene-responsive genes were defined as genes that were at least two-fold differentially expressed in wild type (T89) upon ACC treatment compared to mock treatment (*q* < 0.01). Gene IDs and expression values in response to ACC and mock treatment are listed in Supplementary Table S3.

Parts of the genes in the ethylene pathway have been shown to respond to ethylene itself on a transcriptional level, creating both negative and positive feedback loops (Konishi and Yanagisawa, 2008; Shakeel et al., 2015; Prescott et al., 2016). The rapid effect of exogenous ACC (after 10 h) on ethylene pathway genes was recently analyzed in *Populus* stems (Felten et al., 2018). In this study, ACC was applied to *in vitro* grown wild type (T89) and two ethylene-insensitive (pro*35S::etr1-1* and pro*LMX5::etr1-1*) *Populus* lines which expressed the *A. thaliana* dominant negative mutant allele *etr1-1* (Love et al., 2009). We mapped the sequencing data that originated from hybrid aspen (*P. tremula* × *tremuloides*) and that was presented in Felten et al. (2018) to the *P. tremula* genome. In accordance with expression data presented in Felten et al. (2018), ACC treatment induced expression of *ACO2, ETR1*, and *ETR2, CTR1* and *EIN3C* in an ethylene signaling-dependent manner (in wild type but not in any of the transgenic lines; Figure 2A). Transcriptional regulation of *ETRs* and *CTR1* upon stimulation of ethylene signaling suggests the existence of regulatory feedback loops in the pathway to achieve either increased sensitivity toward ethylene or suppression of the incoming signal depending on the stoichiometric balance between the level of ethylene and the abundance of these negative regulators of ethylene signaling. Consistent with results from Felten et al. (2018), expression of 27 *ERFs* (approximately 16% of all *ERFs*) was significantly altered under enhanced ethylene signaling in response to ACC application.

To study the role of ethylene-mediated transcriptional reprogramming for each phase of wood formation, we extracted stem expression profiles for ACC-responsive genes from the AspWood database (Figure 2B). As a criterion for such genes (from now on called “ethylene-responsive genes”) a two-fold ACC-triggered gene induction/suppression in the ethylene-insensitive trees compared to wild type was used. We observed that 84% of all ethylene-responsive genes have expression profiles in AspWood (Supplementary Table S3). They can be divided on the basis of hierarchical clustering into five clusters that show enhanced transcription in one particular phase (Clusters II-V) or in several phases (Cluster I) of wood formation (Figure 2B). Cluster I, which includes genes with low expression during SCW formation, is enriched with EamA-like transporters, GTPase activating proteins, NAC TFs and peroxidases (see Supplementary Table S3). Among these genes we identified a homolog of *ANAC074* which in *A. thaliana* has been linked to SCW thickening, especially in fibers (Ko et al., 2007). Expression of Cluster II genes showed a peak in the P/C and encode for example ABC transporters, cytochrome P450 proteins, transferases and WRKY and GRAS TF family members. Expression of Cluster III genes was highest during xylem expansion and encode for proteins involved in transcription (e.g., members of the MYC, ERF, WRKY, and MYB TF families) but also several cell wall modifying enzymes (e.g., pectin lyases and xyloglucan endotransglucosylases). Cluster IV, showing the highest gene expression during SCW formation, contained the smallest number of ethylene-responsive genes. These did not show significant enrichment of any protein classes, but contained for instance several immunity-associated genes (e.g., *MAPK*s, *WRKY28*) and peroxidase, UDP-glycosyltransferase85A2 and tetratricopeptide repeat protein genes. Cluster V was comprised of genes encoding transporter and stress-associated proteins (e.g., cytochrome P450, peroxidases) with highest expression in the CD zone. On the basis of this data, we suggest that ethylene-mediated transcriptional reprogramming occurs in all developmental zones. This is in line with the known effects of ethylene on cambial activity (occurring in zone P/C), xylem cell expansion (occurring in the expansion zone) and xylem maturation (Love et al., 2009; Felten et al., 2018).

### *EIN3D* and several *ERFs* are hubs in the wood transcriptome

Several TF families fulfill central roles during secondary growth and wood formation (Duval et al., 2014; Liu et al., 2015; Taylor-Teeples et al., 2015; Sakamoto et al., 2016). To study the importance of the *Populus* EIN3s and ERFs in these processes, we extracted network centrality/connectivity parameters of each annotated gene (“node”) in the AspWood dataset, including the following three parameters: betweenness (BTW), closeness (Cl), and degree (using a default correlation threshold of five) (Sundell et al., 2017). The degree represents the amount of direct correlations of a gene and thus serves as an indicator of the number of other genes with the same (positive correlation) or opposite (negative correlation) expression profile. The BTW and Cl instead give information about the importance of a gene for structure and organization within the network. The BTW serves as a measure of how connected the gene of interest is in the network; i.e., how often a gene is part of the shortest path between other genes. As highly co-expressed genes are more likely to be co-regulated, the BTW serves as an indicator for the involvement of a gene of interest as a regulator of transcription of gene subsets within the network. The Cl can be used as proxy for the distance of a gene of interest to other genes in the network and is calculated as the reciprocal sum of the distances that need to be taken to connect one gene to the others. Nodes (in our case genes) can be divided into four main categories: “center nodes,” highly connected within the network, indicated by a high BTW and Cl and a high degree; “connecting nodes,” low degree but high BTW and Cl indicating their role in connecting subsets of the network; “monopole nodes,” high BTW, but low Cl and degree, typically the only connection between several genes in a small gene module; and “edge nodes,” low BTW and degree, which often are poorly connected genes with little contribution to the network structure. Of particular interest are highly connected genes (“hubs”) as these are likely to reveal important regulators of developmental switches. Hubs are characterized in general by a high BTW but not necessarily a high Cl or degree (Valente et al., 2008). We decided to only focus on the top 20% genes characterized in AspWood (*n* = 2,777), ranked according to their BTW, and defined them as hubs in the current study (Supplementary Table S4). GO term analysis revealed that these hubs are enriched with transporters, potentially involved in membrane organization, ion trafficking and nutrient exchange. SNPs (single nucleotide polymorphisms) in five hubs (*Potri.001G096900, Potri.001G080400, Potri.005G237900, Potri.008G112300, Potri.018G127100*) were previously shown to associate with holocellulose and syringyl lignin content in *Populus* (Porth et al., 2013), supporting the importance of our selected hubs in the gene network underlying wood formation. Among our selected hubs we identified 221 TFs belonging to, among others, the NAC, MYB, C2H2, bZIP, bHLH, ERF, and TALE TF families (Supplementary Table S4). These hub TFs also include homologs of TFs with known functions during SCW formation, such as *SND2* (*SECONDARY WALL-ASSOCIATED NAC DOMAIN PROTEIN 2*; Hussey et al., 2011), *MYB46* (McCarthy et al., 2009; Ko et al., 2014) and *HDG11* (*HOMEODOMAIN GLABROUS 11*; Xu et al., 2014). Furthermore, we identified 21 *Populus* homologs of *A. thaliana* TFs that have been shown to bind to promoters of cellulose, xylan and lignin biosynthesis genes (Supplementary Table S4; Taylor-Teeples et al., 2015). One of them, PtMYB3 (Potri.001G267300), was also shown to bind to promoters of SCW-related genes in *Populus* (Zhong et al., 2013), again validating our selected hubs' importance in wood formation.

**Figure 3:**
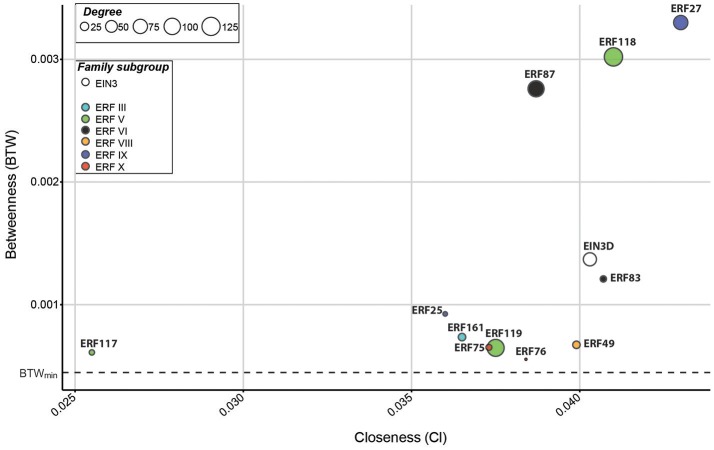
EIN3D and ERFs function as hubs in the wood transcriptome. The graph shows betweenness (BTW) and closeness (Cl) values of *EIN3* and *ERFs* expressed in AspWood (defined in Sundell et al., 2017). Circle size corresponds to their degree. All genes with expression profiles in AspWood were ranked according to their BTW, and the top 20% genes (n, 2,777) were defined as hub genes (listed in Supplementary Table S4). Dotted lines mark the minimum BTW value (0.000447) calculated for a hub gene.

In this network analysis, *EIN3D* and 11 *ERFs* were found among the hubs (Figure 3). Since our aim was to identify TFs that function as central nodes inside the wood transcriptome, we focused on those with a high connectivity. Taken into account their BTW, but also Cl and degree, we focused on eight *ERFs* (*ERF27, ERF49, ERF75, ERF76, ERF83, ERF87, ERF118*, and *ERF119*) for further analysis. Based on their network centrality parameters, *ERF27, ERF87, ERF118, ERF119*, and *EIN3D* were highly connected with other genes in the network, suggesting potential roles as master regulators of gene expression during wood formation. *ERF49, ERF75, ERF76*, and *ERF83* might instead function as mediators of gene expression between diverse gene subsets. Interestingly, only *ERF49, ERF75, ERF76* were responsive to ACC treatment (Figure 2A). Furthermore, *ERF75* carries the EIN3-binding motif (TEIL motif) in its 2 kb promoter, suggesting that it acts directly downstream of EIN3 in ethylene signaling (Felten et al., 2018). In conclusion, the network centrality analysis resulted in identification of *EIN3D* and eleven *ERF*s as *Populus* TFs that are likely to control transcriptional changes during secondary growth. This is supported by recent finding on an *ERF118* homolog in *P. simonii* × *nigra* (*PsnSHN2*) in controlling the expression of SCW-related TFs and modulating secondary cell wall properties (Liu et al., 2017). Functional evidence exists also for *Populus* ERF76 in connection to abiotic stress response (Yao et al., 2016, 2017). To our knowledge, these are however the only functionally characterized EIN3/ERF hubs so far, highlighting the need for future functional studies of these master regulators and their downstream targets.

### The *EIN3D* co-expression gene module reveals several TFs as potential novel regulators of wood formation

**Figure 4:**
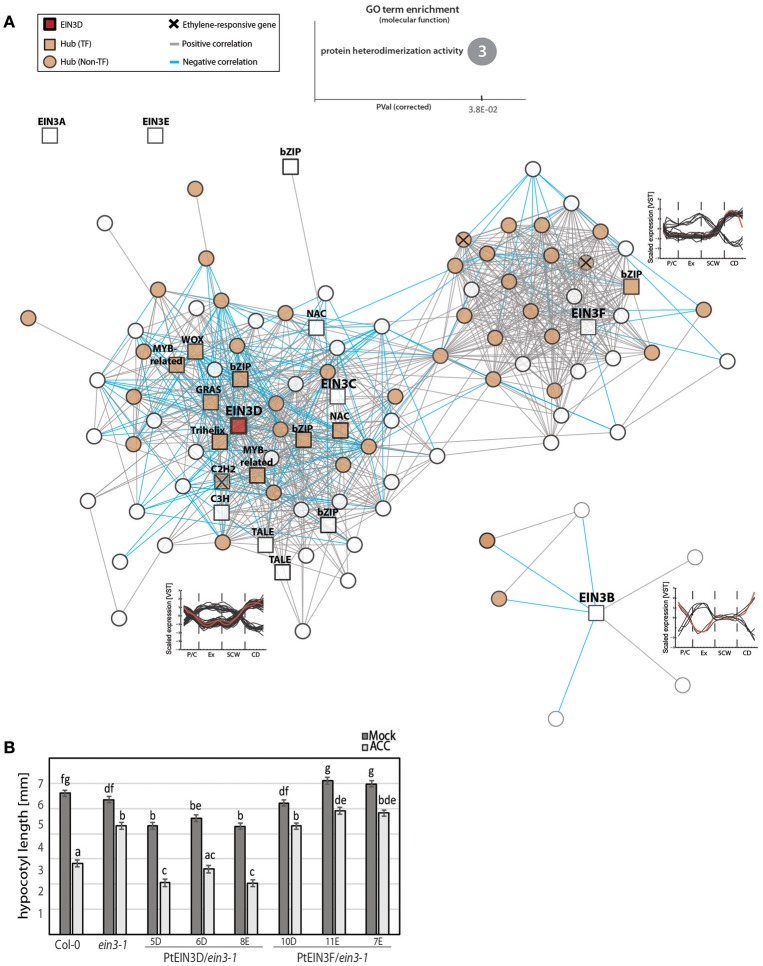
EIN3 isoforms are co-expressed with TFs involved in transcriptional regulation and secondary cell wall biosynthesis. **(A)** Co-expression gene modules (threshold of five) of *EIN3B, EIN3C, EIN3D*, and *EIN3F*. *TFs* are shown as squares, while all *non-TFs* are represented by circles. Hubs are indicated by orange filling. Positive correlation between two genes is represented by gray lines (edges), negative correlations are indicated by blue edges. Ethylene-responsive genes shown in Figure 2C are marked with a cross (see Supplementary Table S5). Line graphs show scaled expression profiles of genes in each modules during wood formation, with red lines depicting the expression pattern of each *EIN3* isoform. GO term enrichment was performed with *Arabidopsis* homologs. Number in the circle indicates number of genes belonging to this GO term found among all 87 *EIN3* co-expressed genes. **(B)** Estimate of hypocotyl length [in mm] with standard error, calculated using a linear effect model. Vernalized seeds were germinated in the dark for 72 h in the presence/absence of 10 μM ACC. Hypocotyl length was determined from 29 to 44 biological replicates (depending on genotype, treatment and experiment) obtained from two independent experiments. Raw data, including number of biological replicates per line, treatment and experiment can be extracted from Supplementary Table S8.

Although the seven *Populus EIN3s* had very similar spatial expression patterns in AspWood, only *EIN3D* passed our selection for hubs in the AspWood dataset. This apparent functional diversification prompted us to study the structure of the co-expression network for the different *Populus EIN3* TFs. Using a co-expression cutoff of five (in accordance with the network analysis used in Sundell et al., 2017), we identified three distinct gene modules that contained genes co-expressed with either *EIN3C* and *EIN3D, EIN3F*, or *EIN3B* (Figure 4A). Among these three gene modules, we found three genes that showed ethylene-dependent transcriptional regulation upon ACC treatment (marked with a cross in Figure 4A), indicating that EIN3s contribute to the transcriptional regulation of ethylene-responsive genes during wood formation. Furthermore, all *EIN3* gene modules were enriched with hubs (marked as orange nodes in Figure 4B), indicating co-expression and thus a potential regulatory function of *EIN3B, EIN3C/EIN3D*, and even *EIN3F* on other key genes during wood formation (Supplementary Table S5). Surprisingly, no *ERFs* were present in any of the *EIN3* gene modules, suggesting that the expression of *ERF*s might not require EIN3s during normal wood formation. It is possible that the control of *ERFs* through EIN3 becomes more prominent under stress, when ethylene levels increase. 14 of the 27 ACC-regulated *ERFs* carried the TEIL motif in their 2 kb promoter indicating a potential regulation of at least part of the *ERFs* through EIN3s upon high ethylene levels (Felten et al., 2018). Also other studies on EIN3-mediated regulation of *ERFs* support the idea of EIN3-mediated *ERF* regulation under stress conditions or exogenous ACC/ethylene application (for recent examples see Chang et al., 2013; Quan et al., 2017).

The largest gene module consists of 64 nodes including *EIN3D*. Representatives of eight different TF families, including EIN3 itself, NAC, bZIP, MYB-related, TALE, WOX, GRAS, C2H2, and C3H, were present in the *EIN3D* gene module (Supplementary Table S5). Among them we found a homolog of VND-INTERACTING 2 (VNI2; AT5G13180) which in *A. thaliana* interacts with VND7 and functions to suppress xylem vessel formation (Yamaguchi et al., 2010) and also NARS1/NAC2 (AT3G15510) shown to be involved in SCW development of seed coat epidermal cells (Voiniciuc et al., 2015). Comparing the *EIN3* co-expressed genes to a publicly available dataset of direct targets of *A. thaliana* EIN3 (Chang et al., 2013), we found ten shared genes of which eight belong to the *EIN3D* gene module (Supplementary Table S5). This result suggests that EIN3D directly controls at least part of its co-expressed genes, including the homolog of *VNI2* (*Potri.003G166500*). Although protein interaction studies with EIN3 and other TFs are still elusive, this result suggests that EIN3D might act upstream or together with VNI2 during wood formation.

In order to asses *Populus* EIN3D functionality in ethylene signaling, we tested its capacity to complement the *A. thaliana* ethylene-insensitive *ein3-1* mutant by expressing it under the control of the 35S promoter (Figure 4B, Supplementary Table S8). We also included EIN3F as a representative of the three *P. trichocarpa EIN3* genes that clustered together with *AtEIL3* which cannot complement *ein3-1* (Supplementary Figure S1G; Chao et al., 1997). Complementation was assessed using the triple response of dark-grown (etiolated) *A. thaliana* seedlings as the phenotypic output (Guzmán and Ecker, 1990) in three transgenic lines for each *Populus EIN3* gene. External application of ACC or ethylene results in shortening and thickening of the hypocotyl and root as well as exaggerated curving of the apical hook (Stepanova and Alonso, 2009), which is diminished in *ein3-1*. Overexpression of *EIN3D* showed consistent ability to complement *ein3-1*, as demonstrated by the effect of ACC in reducing hypocotyl length of the transgenic lines to a length similar or smaller than in the wild type. Overexpression of *EIN3F* did not complement *ein3-1*. Hence, our data supports that the *Populus* EIN3D can rescue the loss of function of *A. thaliana EIN3* and is therefore functional in the process of ethylene controlled hypocotyl elongation. However, the function of EIN3D during wood formation remains to be elucidated.

### *ERF* gene modules comprise genes associated with SCW biogenesis

**Figure 5:**
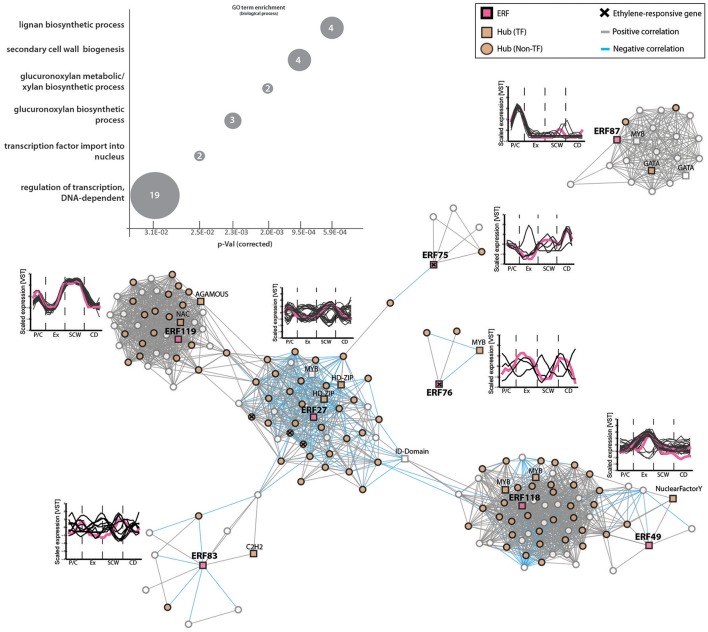
*ERF* gene modules are associated mainly with pectin, lignan and xylan biogenesis during secondary growth. Co-expression gene modules of *ERF27, ERF75, ERF76, ERF83, ERF87, ERF118, ERF119* (threshold of five). *TFs* are shown in squares, while all *non-TFs* are represented by circles. Hubs are indicated in orange. Positive correlation between two genes is represented by gray lines (edges), negative correlations are indicated by blue edges. Ethylene-responsive genes (shown in Figure 2C) inside each gene module are marked with a cross (see Supplementary Table S6). Line graphs show expression profiles (scaled) of genes in each module during wood development. Pink lines mark *ERF*s inside each gene module. GO term enrichment was performed with *A. thaliana* homologs (Supplementary Table S6). The numbers indicate number of genes belonging to this GO term found among all 143 *ERF* co-expressed genes.

We selected eight out of 11 *ERF*s that were found as hubs for further analysis (*ERF27, ERF49, ERF75, ERF76, ERF83, ERF87, ERF118, ERF119*). We investigated co-expression gene modules of these hubs to elucidate their role in wood formation (Figure 5). GO term enrichment analysis with the *A. thaliana* homologs of the co-expressed genes showed a significant enrichment of genes associated to SCW biogenesis (e.g., genes encoding for lignan and xylan biosynthesis). Similar to our results, the AP2/ERF *Ii049* from *Isatis indigotica* was recently linked to lignan biosynthesis (Ma et al., 2017). We also observed that each of these *ERF*s is part of a gene module which contains several other hubs, further supporting their central function during wood formation.

The *Populus ERF27* was part of the largest gene module containing co-regulated genes with strong expression in the phloem and during SCW formation or, *vice versa*, low during these phases and high in the cambium-xylem expansion phase and during xylem maturation (Figure 5). The module contained a few genes that were connected to carbohydrate household, such as *UDP-glycosyltransferase88A1* and *SUC2 (ARABIDOPSIS THALIANA SUCROSE-PROTON SYMPORTER 2)*, but also several oxidative stress- and salt stress-related genes. Interestingly, the expression of *ERF27* correlated negatively with the expression of a homolog of *A. thaliana HOMEOBOX GENE 8* (*AtHB8*; Supplementary Table S6). AtHB8 has been shown to control procambial cell specification in *A. thaliana* leaves (Donner et al., 2009), and overproduction of AtHB8 stimulates cambial cell proliferation and xylem differentiation (Baima et al., 2001), thus linking ERF27 to processes occurring in the vascular cambium and differentiating xylem.

The *Populus ERF118* and *ERF49* were part of one gene module but not directly connected. All genes in this module had their highest expression in the xylem expansion phase. The module was enriched in genes encoding primary cell wall modifying enzymes, such as a xyloglucan endotransglycosylase (*Potri.013G005700*), a pectin methylesterase (*Potri.002G202500*) and a pectin lyase (*Potri.014G117100*) (Supplementary Figure S6). Hence we propose that genes in the *ERF118/ERF49* module are associated with primary wall modifications during xylem cell expansion. *ERF119* on the other hand was strongly induced during SCW formation, and the *ERF119* co-expressed genes were associated for instance with lignan [pinoresinol reductase (*Potri.003G100200*)] and xylan biosynthesis (galacturonosyltransferases *Potri.001G416800* and *Potri.011G132600*).

In accordance with the *EIN3* modules lacking *ERFs*, the *ERF* modules did not contain any *EIN3* genes either (Supplementary Table S6), further supporting the hypothesis that EIN3s might only control *ERFs* that are responsive to high ethylene levels. In addition, we did not identify shared nodes between the *EIN3* and *ERF* gene modules (Supplementary Table S5), supporting diverse functions for these TF families during wood formation.

### Ethylene-responsive TFs are co-expressed with immune response genes during wood development

**Figure 6:**
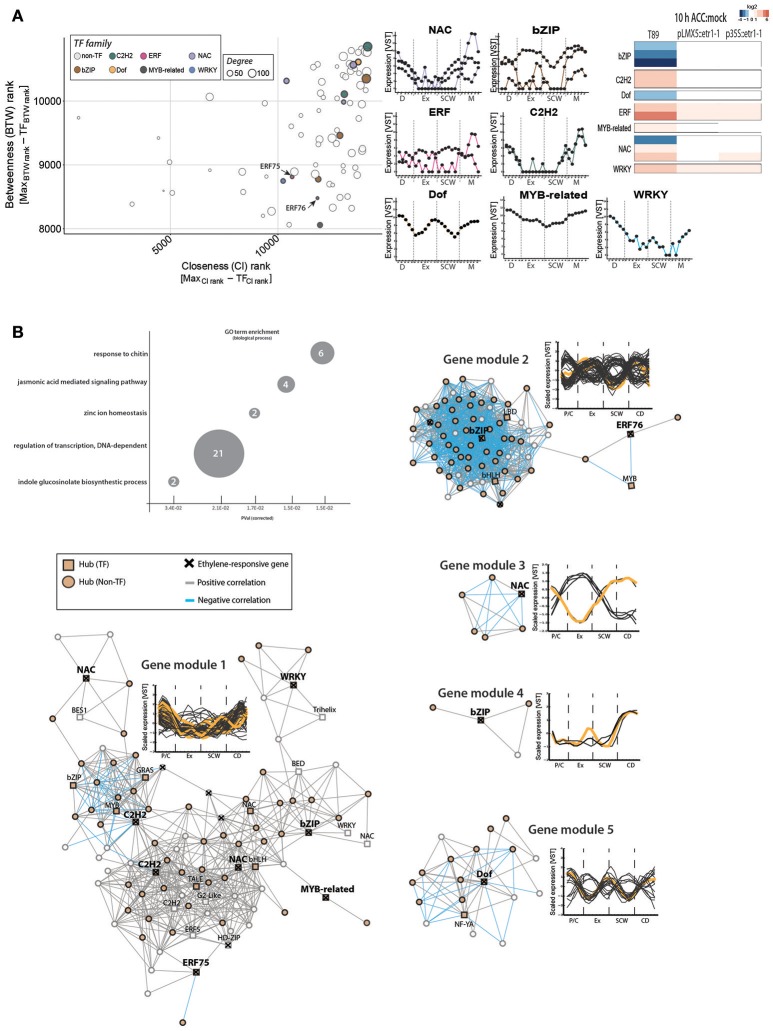
Expression of ethylene-responsive hub *TF*s is associated with immunity-related phytohormone signaling during wood formation. **(A)** Gene centrality parameters and expression profiles of ethylene-responsive hub *TF*s. ACC-triggered expression values in wild type and two ethylene-insensitive trees were obtained as described in Figure 2. **(B)** Co-expression gene modules (threshold five) of ethylene-responsive *TF*s. Ethylene-responsive genes inside each gene module are marked with a cross (see Supplementary Table S7). GO term enrichment was performed with *A. thaliana* homologs. Number indicate number of genes belonging to this GO term found among all 150 co-expressed genes.

Only two *Populus ERFs* among the selected *ERF* hubs (*ERF75* and *ERF76*) were ethylene responsive (Figure 5). In order to identify additional molecular players that connect ethylene signaling and wood formation, we enlarged our network analysis and included all TFs among the ethylene-responsive genes shown in Figure 2B, independent of their TF family background (Figure 6). We found three *bZIP*, three *NAC*, two *C2HC*, one *Dof*, one *Myb-related*, and one *WRKY* as hubs which displayed ethylene signaling-dependent expression in response to ACC (Figure 6A). One of the ethylene-responsive *NAC* TFs is a homolog of *ANAC012* (*Potri.002G037100*), which is a negative regulator of secondary wall deposition in xylem fibers (Ko et al., 2007). A second *NAC* is a homolog of *ANAC047* that has been shown to act downstream of the EIN2-EIN3 signaling cascade during leaf senescence (Kim et al., 2014). While the TEIL motif was not found in the promoter region (2 kb upstream the start codon) of the *Populus* homolog of *ANAC012* nor *ANAC047*, promoter regions of two other ethylene-responsive TFs [*ERF75* and *ANAC100* (*Potri.017G086200*)] did contain a TEIL motif (Felten et al., 2018). These results support EIN3-mediated transcriptional regulation of a subset of the ethylene-responsive TFs and thus their potential function in wood formation in an ethylene-dependent manner.

Expression of eight ethylene-responsive TF hubs was co-regulated, with highest expression in phloem/cambium cells and during CD (gene module 1; Figure 6B; Supplementary Table S7). GO term enrichment tests indicated that the *A. thaliana* homologs of co-expressed genes are involved in immune responses against chitin (for example WRKY-encoding *Potri.006G109100, Potri.015G099200*), phytohormone-mediated responses (jasmonic acid response; auxin-related indole glucosinolate biosynthesis), transcriptional regulation and zinc ion homeostasis. Jasmonates have previously been connected to secondary growth in *A. thaliana* (Sehr et al., 2010). Also, treatment with jasmonates to aspen plantlets induced the formation of tyloses, which are occlusions of xylem vessels that serve as a barrier against pathogens (Leśniewska et al., 2017). This study also showed that combined exogenous application of ACC and jasmonic acid triggered tyloses formation in an ethylene signaling-dependent manner. In wild type trees, ACC treatment led to suppression of all *bZIP, Dof* and one *NAC* TFs, suggesting that these are negative regulators of ethylene-mediated transcriptional reprogramming. Indeed, one *bZIP* (*Potri.005G243400*, gene module 2) and the *Dof* TF (*Potri.004G038800*; gene module 5) showed mainly negative correlation with other hubs in their co-expression gene modules. The bZIP TF is a homolog to the *A. thaliana* FD, a flowering associated regulator (Abe et al., 2005), and its expression showed a low sharp peak during SCW formation. The importance of gene regulation by these ethylene-regulated *TF*s for wood development remains unanswered. However, we found several shared nodes between gene module 1 and the *EIN3D*-associated gene module (Supplementary Table S7), encoding for example for 6-BETA-TUBULIN (*Potri.009G067100*) or a homolog of mannanase regulator *AtBZIP44* (Iglesias-Fernández et al., 2013), pointing toward a link between EIN3D and the ethylene-regulated TFs on transcriptional regulation during secondary growth.

## Conclusion

Unraveling transcriptional regulation mechanisms by phytohormones is key for understanding numerous aspects of the plant life cycle. Our study identified the homologs of ethylene biosynthesis and signaling components in *Populus* and their expression profiles during secondary growth. Co-expression network analysis of the wood transcriptome revealed a plethora of biological processes, such as lignan, xylan and pectin biosynthesis, which were transcriptionally associated with *EIN3D* and several *ERFs*. *EIN3D* and 11 *ERFs* were identified as hub TFs in a tissue-specific manner. Notably, we identified EIN3D and its' co-regulated TFs as potential transcriptional master switches during wood formation, and ERF118 and ERF119 having a potential role in regulating xylem expansion and SCW formation, respectively. Upcoming research projects focusing on the function and downstream targets of these TFs are predicted to significantly broaden our understanding of the role of ethylene in wood formation and to highlight possibilities to utilize ethylene pathway genes in forest biotechnology and tree breeding practices.

## Materials and methods

### Phylogenetic analyses of genes putatively related to ethylene signaling and biosynthesis

The gene family members of ethylene receptor, *CTR1, EIN2, EIN3*, and *ERF* genes of *A*. *thaliana, P*. *trichocarpa* and *P*. *abies* were extracted from the Plant Genome Integrative Explorer resource (PlantGenIE.org; Sundell et al., 2015; Gene Family tab). In order to find homologous gene models putatively placed in separate gene families and *P*. *abies* full-length transcripts represented by low quality gene models, BLAST searches were also performed against PlantGenIE genome and transcriptome databases. Subsequently, the identified gene models and transcripts were aligned in Clustal Omega with default parameters (Sievers et al., 2011) and aberrant sequences were removed. The phylogenetic trees were created using the Galaxy platform (Goecks et al., 2010) hosted at PlantGenIE.org. The Galaxy workflow utilized the MUSCLE v3.8.31 program (maximum number of iterations: 16) for multiple alignment, and PhyML 3.1 (substitution model: WAG, aLRT test: SH-like, tree topology search operation: Nearest Neighbor Interchange) and Tree Vector programs for building and drawing phylogenetic trees, respectively.

### Cloning and mutant generation

In order to ascertain whether *Populus* homologs of *A. thaliana* EIN3 could rescue the triple response of the *A. thaliana ein3-1* mutant, genomic DNA was extracted from *P. trichocarpa* leaves using the E.Z.N.A. kit (Omega). Full length *Populus EIN3D* and *EIN3F* was amplified (primers listed in Supplementary Table S9) and cloned into pENTR/D-TOPO (pENTR Directional TOPO Cloning Kits, Invitrogen) and thereafter transferred to pB7GW2D through Gateway Recombination (LR Clonase II, Invitrogen). The resulting plasmids (p35S:*PtEIN3*) were transformed into *Agrobacterium tumefaciens* GV3101 (pMP90) followed by transformation of *ein3-1* through floral dip (Clough and Bent, 1998). Homozygous lines were selected based on Basta resistance (50 μM). The triple response assay was performed with three homozygous lines.

### Triple response assay

Transgenic lines were germinated for 72 h in the dark on Murashige-Skoog media that was either supplemented with 10 μM ACC or without (Alonso-Stepanova Laboratory Protocols)^1^. Hypocotyl and root lengths were measured for each seedling in the presence and absence of ACC (root length not shown, but phenotypes are consistent in both tissues). Mean, standard error and *p*-values were calculated from 29 up to 44 biological replicates (depending on genotype and treatment) using a linear effect model (lme function in *limma*), with genotype and treatment as fixed effects. The *multcompView* package was used to assign significance letters using a *p*-value cutoff 0.01. Supplementary Table S8 includes all raw data, number of replicates per genotype, treatment and experiment and output (mean, standard error, *p*-values and significance letters) calculated from the linear model.

### RT-qPCR analysis

Transgene expression was quantified from three pools of five seedlings harvested from control plates. Total RNA was isolated according to instructions using the Aurum™ Total RNA Mini Kit (Bio-Rad) and a DNAse treatment was performed using the Ambion® DNA-free™ DNA Removal kit (Thermo Fisher Scientific). RNA was quantified and cDNA was synthesized using the iScript cDNA Synthesis Kit. Real-time quantitative PCR (qPCR) was performed on five times diluted cDNA template using a Bio-RAD CFX96 Real Time System with SYBR® Green Mastermix (Bio-Rad) and 5 pmol concentrated primers. PCR conditions were as follows; 3 min initial denaturation at 95°C, followed by 39 cycles of denaturation at 95°C for 10 s, primer annealing at 58°C for 10 s, and a 20 s extension step at 72°C. *AtEF1*α (*At5G60390*) was used as a reference gene. ΔCt values were calculated by subtracting average *EF1*α Ct value from corresponding Ct value. Relative expression levels were calculated using the formula 2^−Δ*Ct*^ for each sample (Supplementary Figure S5). Primers are listed in Supplementary Table S9.

### RNA-SEQ data analysis

Description of tree growth conditions, experimental setup for treatment with ACC and RNA extraction and library preparation procedure can be found in Felten et al. (2018). Briefly, RNA was extracted from whole stems from wild type (T89) and two ethylene-insensitive trees (pLMX5::*etr1-1* and p35S::*etr1-1*). *In vitro*-grown plants were allowed to grow until a height of approximately 8 cm. For the treatment, 100 μM ACC or water was applied on top of the medium. Stem material was pooled from six plants per treatment and genotype 10 h after application of either ACC or water. Frozen stems were ground and used for RNA extraction using the CTAB method and lithium chloride precipitation. DNA was removed using *DNAfree*™ (Ambion), left-over RNA was cleaned using the Qiagen MinElute kit and sent for library generation and paired-end Illumina sequencing to SciLifeLab (Science for Life laboratory, Stockholm, Sweden). The sequencing data is available from the European Nucleotide Archive under the accession number ERP012528. Quality assessment of raw sequence data, including removal of ribosomal RNAs and sequencing adapters and quality trimming of sequences was performed as described in Felten et al. (2018). Read pairs that passed the quality assessment were mapped to the latest *P. tremula* genome sequence retrieved from “PopGenIE” (www.popgenie.org). We chose the *P. tremula* genome since its genetic background is most similar to hybrid aspen (*P. tremula* × *tremuloides*; Hamzeh and Dayanandan, 2004), which was the species used for the ACC application experiment. Reads were transformed into a count per gene per library using HTSeq (Anders et al., 2015). Statistical data analysis was performed in R (version 3.2.2) using *EdgeR*. First, reads with less than 10 counts in at least one library were excluded from the dataset. Gene counts were normalized based on a calculated normalization factor (function calcNormFactors in the R package *edgeR*). Count data was log-transformed (voom function in R package *limma*) to obtain log2 counts per million. The lmfit function in the *limma* package was used to fit a mixed linear effect model (with genotype:treatment as fixed effects and biological replicate as random effect) to the log2 gene expression values and variance shrinkage was applied using the eBayes function in *limma* before calculating *p*-values. An FDR (false discovery rate) adjusted *p*-value (*q*-value) cutoff of 0.01 was used to extract differentially expressed genes. In order to compare the effect of ACC application in the ethylene-insensitive trees to wild type trees, we set a cutoff of two-fold ACC-triggered expression difference between wild type and both ethylene-insensitive trees and defined the genes that passed this criterion as “ethylene-responsive genes.” Heatmaps were generated with the *pheatmap* package in R.

### Co-expression network analysis

Obtained RNA-Seq reads from stem sections of four trees were aligned to the *P. trichocarpa* genome and normalized using a variance stabilizing transformation (VST). Genes with a VST > 3 in at least two samples from at least three out of four trees were considered as differentially regulated. All samples (stem sections) were clustered using Euclidean distance and all genes were scaled and clustered using Pearson correlation. The co-expression network was performed using mutual information (MI) and context likelihood of relatedness (CLR) algorithm. The co-expression network is purely based on gene expression profiles irrespective of the cell type. Selection of hubs in the transcriptome during secondary growth was performed on the basis of the BTW rank obtained for each gene in AspWood (described in Sundell et al., 2017). The BTW rank was calculated as follows: first the betweenness was calculated (= number of cases in which a node lies on the shortest path between all pairs of other nodes) and afterwards calculated values were sorted in ascending order (highest betweenness value was associated to 1). The Cl rank (= reciprocal of the sum of distances to all other nodes) was calculated in the same way. All genes present in AspWood were ranked according to their BTW rank and the top 20% genes (*n* = 2,777) were defined as “hubs.” TFs among the hubs were selected according to their characterization in AspWood and presented in Figure 3, Supplementary Figure S4 with their centrality parameters (BTW, Cl and degree). In accordance to Sundell et al. (2017), all presented gene co-expression networks (Figures 4–6) are generated using a co-expression threshold of five. Composition of each gene module was analyzed using a build-in function of the AspWood database.

### GO term analysis

GO terms (in this case significantly enriched PFAMs) that were used to describe clusters of ethylene-responsive genes in Figure 2B were extracted from AspWood (listed in Supplementary Table S3). Cluster-based gene selection was done according to their expression profile in AspWood (defined by Clusters A-H in Aspwood). GO term analysis shown in Figures 4–6 were performed with *A. thaliana* homologs. Significantly enriched GO terms were extracted from “AtGenie” (http://atgenie.org/enrichment) using a *p*-value cutoff of 0.05 (listed in Supplementary Tables S5–S7).

## Author contributions

CS performed co-expression network analysis and all bioinformatic analyses. BW performed the complementation experiment. SJ-L performed the phylogenetic analyses. ND helped with the RNA-Seq analysis. BS contributed to the complementation experiment. CS, BW, JF, and HT analyzed and discussed the data. The manuscript was written by CS and HT with contributions from all coauthors.

### Conflict of interest statement

The authors declare that the research was conducted in the absence of any commercial or financial relationships that could be construed as a potential conflict of interest. The reviewer MB declared a past co-authorship with one of the authors HT to the handling Editor.

## Footnotes

^1^Alonso-Stepanova Laboratory Protocols. Available online at: http://www4.ncsu.edu/~jmalonso/Alonso-Stepanova_ACC.html (Accessed Sept 20, 2017).

## Supplementary material

Supplementary Figure S1 Phylogenetic analysis of genes involved in ethylene biosynthesis **(A–C)**, perception and signaling **(D,E)**, transcriptional reprogramming **(F,G)** and regulation **(H)** in *A. thaliana* (*At*; brown) and the two woody species *P. trichocarpa* (*Pt*; black) and *P. abies* (*Pa*; gray). Exact gene identities can be extracted from Supplementary Table S1.

Supplementary Figure S2 Protein alignment of CTR1 isoform(s) of *A. thaliana (At), P. trichocarpa (Potri), P. abies (MA), Salix purpurea (Sapur)*. Kinase domain is labeled according to Huang et al. (2003).

Supplementary Figure S3 Expression pattern of analysis of genes involved in ethylene biosynthesis **(A–C)**, perception and signaling **(D,E)**, transcriptional reprogramming **(F,G)** and regulation **(H)** during secondary growth in *P. trichocarpa*. Gene expression data was extracted from the AspWood database (Sundell et al., 2017). Each data point represents expression values in cryosections of zones labeled as P/C, Phloem/Cambium cells; Ex, Expanding xylem; SCW, Secondary cell wall formation; CD, Cell death. Raw data used for this figure can be found in Supplementary Table S2.

Supplementary Figure S4 TF hubs from the AspWood network. Centrality parameters of TF hubs (BTW, Cl and degree, listed in Supplementary Table S4). Colors indicate TF family. The plant TF database (v4.0) was used to identify TF families. Families that are not present in top 20% genes are: ARR-B, BBR-BPC, BES1, CAMTA, E2F, FAR1, GeBP, HB-PHD, HB-others, HRT-like, LFY, LSD, NF-XI, NZZ/SPL, RAV, SAP, VOZ, Whirly.

Supplementary Figure S5 Transgene expression levels in 35S::PtEIN3D*/ein3-1* and 35S::PtEIN3F*/ein3-1*. Each bar represents the mean of three pools of seedlings ± SD, with each pool consisting of five seedlings per tested line.

Supplementary Table S1 Gene identities and abbreviations used for phylogenetic analysis of gene families in the ethylene pathway.

Supplementary Table S2 Expression values for genes in the ethylene pathway during secondary growth.

Supplementary Table S3 Expression values of ethylene pathway and ethylene-responsive genes upon ACC treatment.

Supplementary Table S4 Identified hubs from “AspWood” co-expression network.

Supplementary Table S5 Structure and assembly of EIN3 co-expression networks.

Supplementary Table S6 Structure and assembly of ERF co-expression networks.

Supplementary Table S7 Structure and assembly of co-expression networks from ethylene-responsive TFs.

Supplementary Table S8 Complementation of ein3-1 mutants by PtEIN3D.

Supplementary Table S9 Primers used in this study.
