# Supplementary figures and images for: Ethylene-Related Gene Expression Networks in Wood Formation
Source: Front Plant Sci. 2018 Mar 14;9:272. doi: 10.3389/fpls.2018.00272 (PMC5861219; doi:10.3389/fpls.2018.00272)

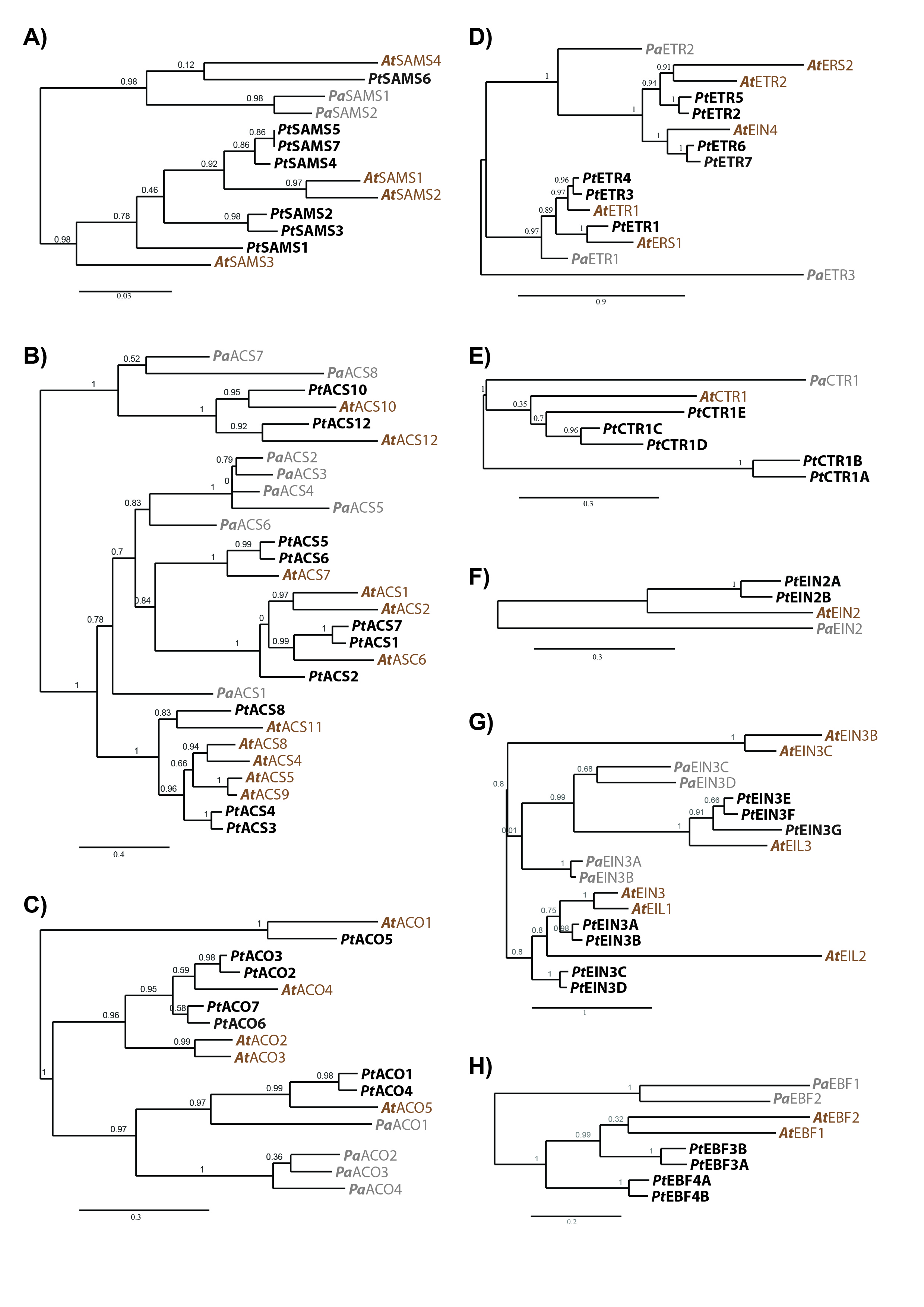

Supplement: Supplementary Figure S1 — Phylogenetic analysis of genes involved in ethylene biosynthesis (A–C), perception and signaling (D,E), transcriptional reprogramming (F,G) and regulation (H) in A. thaliana (At; brown) and the two woody species P. trichocarpa (Pt; black) and P. abies (Pa; gray). Exact gene identities can be extracted from Supplementary Table S1. [file Image1.JPEG]

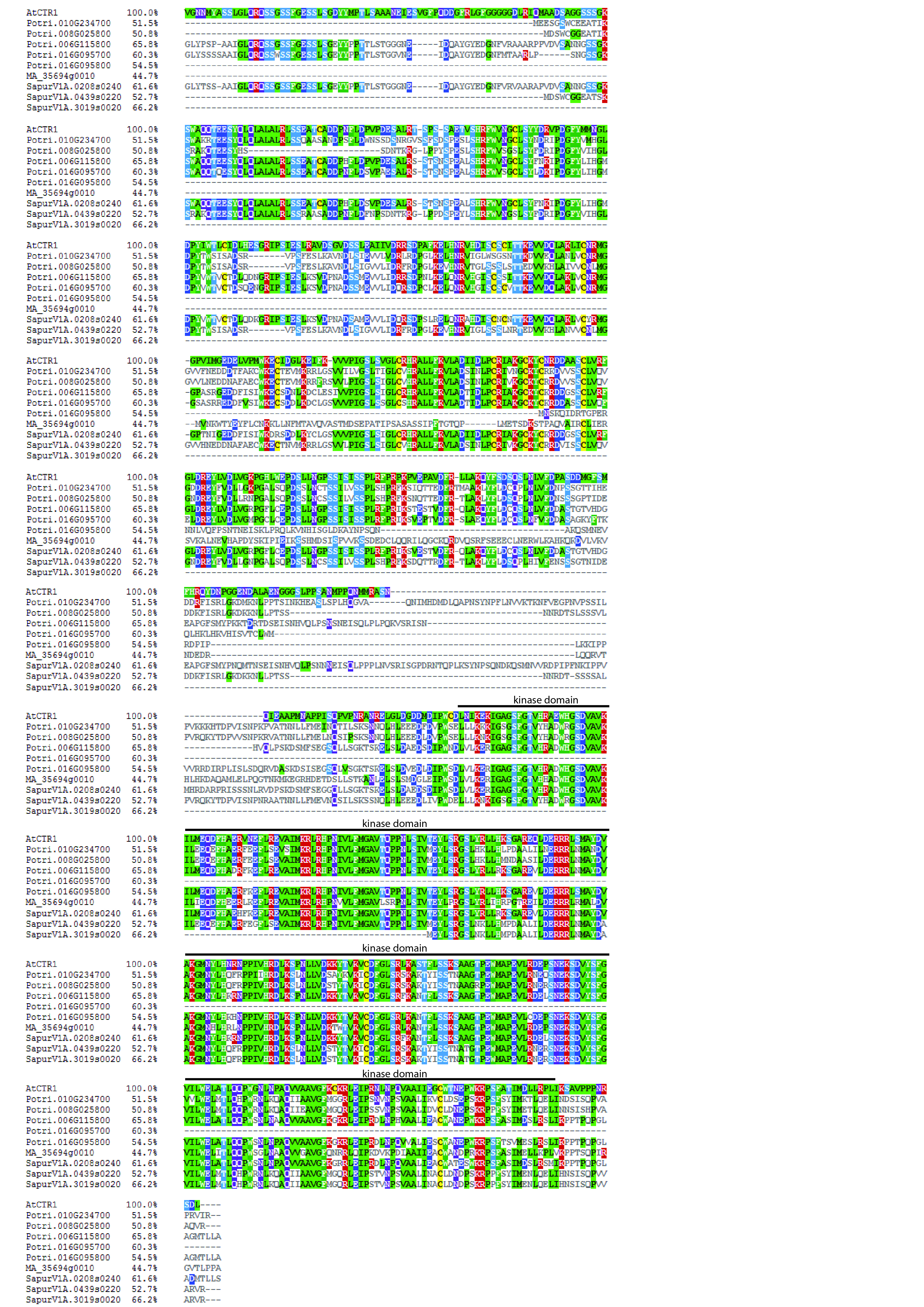

Supplement: Supplementary Figure S2 — Protein alignment of CTR1 isoform(s) of A. thaliana (At), P. trichocarpa (Potri), P. abies (MA), Salix purpurea (Sapur). Kinase domain is labeled according to Huang et al. (2003). [file Image2.JPEG]

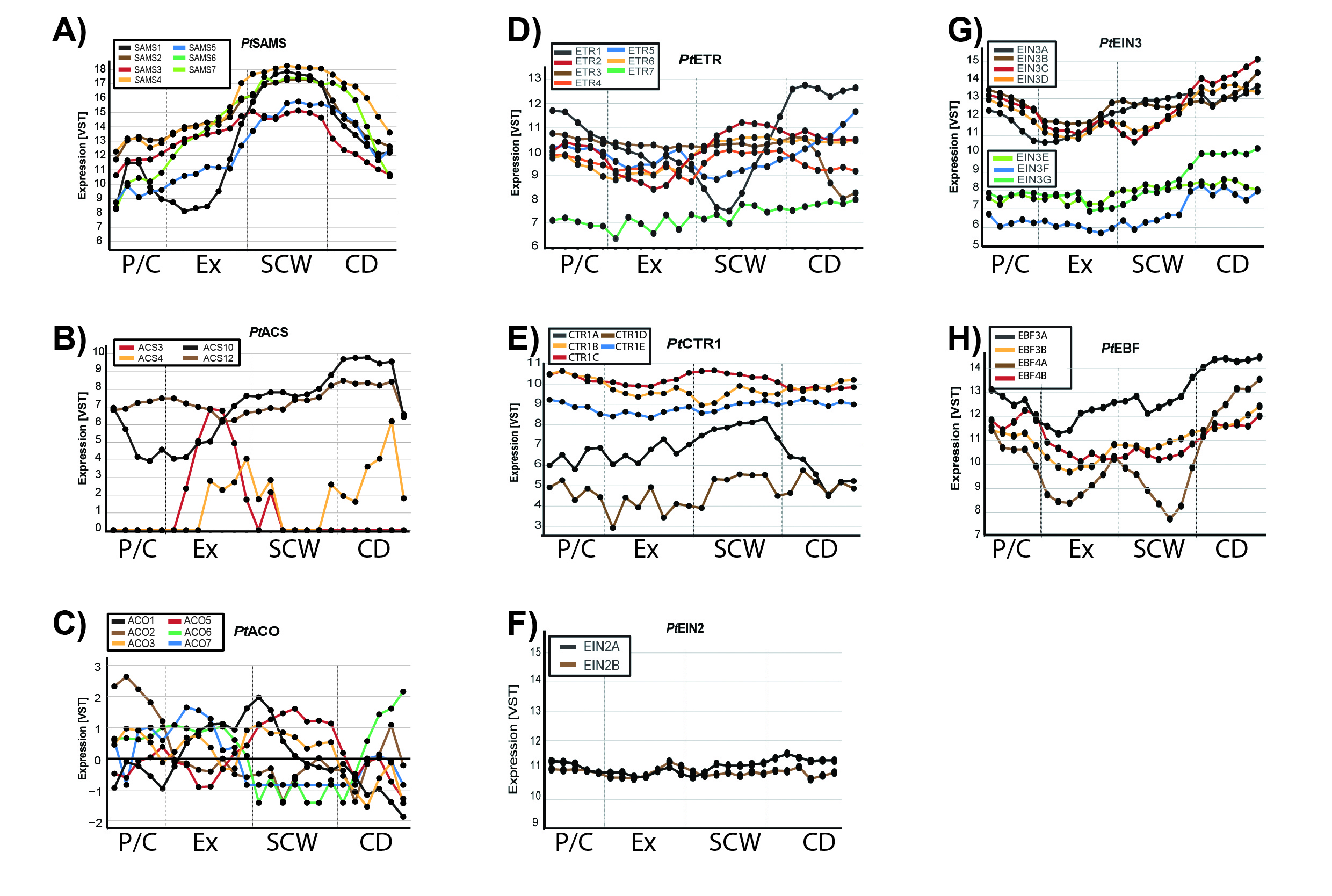

Supplement: Supplementary Figure S3 — Expression pattern of analysis of genes involved in ethylene biosynthesis (A–C), perception and signaling (D,E), transcriptional reprogramming (F,G) and regulation (H) during secondary growth in P. trichocarpa. Gene expression data was extracted from the AspWood database (Sundell et al., 2017). Each data point represents expression values in cryosections of zones labeled as P/C, Phloem/Cambium cells; Ex, Expanding xylem; SCW, Secondary cell wall formation; CD, Cell death. Raw data used for this figure can be found in Supplementary Table S2. [file Image3.JPEG]

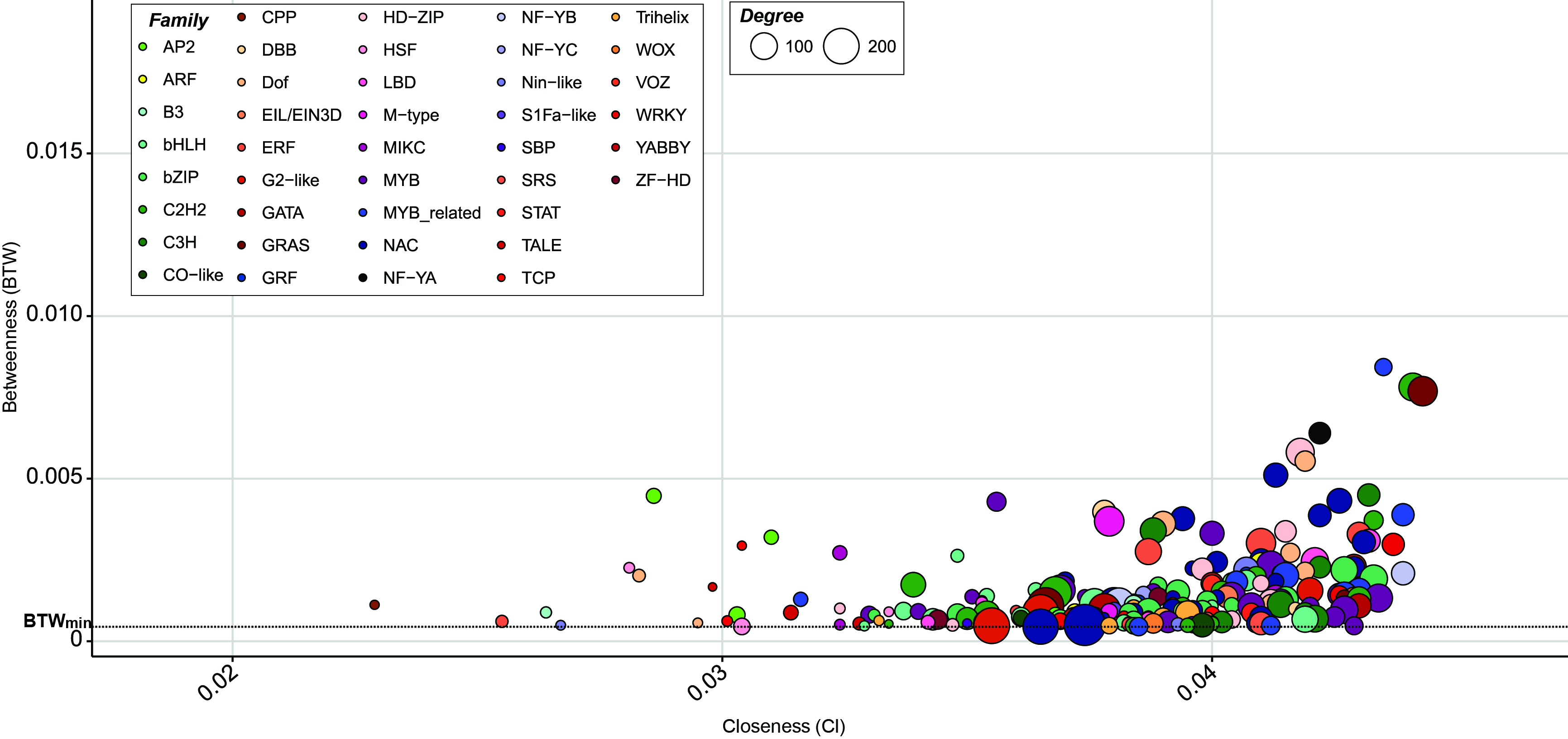

Supplement: Supplementary Figure S4 — TF hubs from the AspWood network. Centrality parameters of TF hubs (BTW, Cl and degree, listed in Supplementary Table S4). Colors indicate TF family. The plant TF database (v4.0) was used to identify TF families. Families that are not present in top 20% genes are: ARR-B, BBR-BPC, BES1, CAMTA, E2F, FAR1, GeBP, HB-PHD, HB-others, HRT-like, LFY, LSD, NF-XI, NZZ/SPL, RAV, SAP, VOZ, Whirly. [file Image4.JPEG]

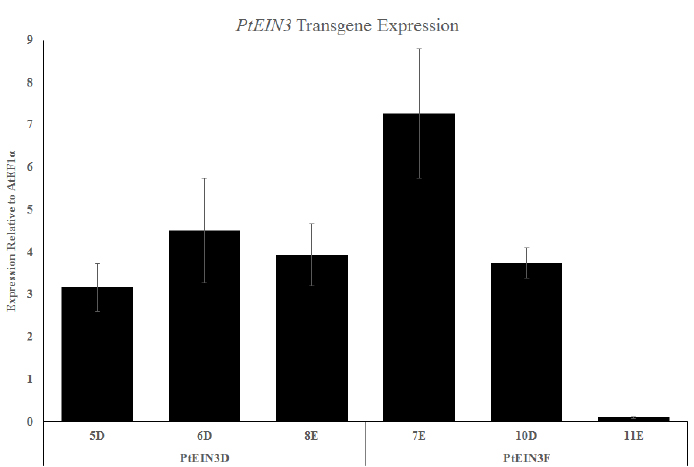

Supplement: Supplementary Figure S5 — Transgene expression levels in 35S::PtEIN3D/ein3-1 and 35S::PtEIN3F/ein3-1. Each bar represents the mean of three pools of seedlings ± SD, with each pool consisting of five seedlings per tested line. [file Image5.JPEG]
